# Longitudinal Associations Between Perceived Parent-Adolescent Attachment Relationship Quality and Generalized Anxiety Disorder Symptoms in Adolescence

**DOI:** 10.1007/s10802-012-9613-z

**Published:** 2012-02-23

**Authors:** Fenna E. A. M. van Eijck, Susan J. T. Branje, William W. Hale, Wim H. J. Meeus

**Affiliations:** Research Centre Adolescent Development, Utrecht University, P.O. Box 80140, 3508 TC Utrecht, The Netherlands

**Keywords:** Adolescence, GAD symptoms, Father-adolescent relationships, Mother-adolescent relationships, Gender differences, Age differences

## Abstract

This longitudinal study examined the direction of effects between adolescents’ generalized anxiety disorder (GAD) symptoms and perceived parent-adolescent attachment relationship quality, as well as the moderating role of gender and age. 1,313 Dutch adolescents (48.5% boys) from two age cohorts of early (*n* = 923, M_age_ = 12 at W1) and middle (*n* = 390, M_age_ = 16 at W1) adolescents completed questionnaires regarding their attachment relationship to parents and GAD symptoms in four waves. Cross-lagged path analyses demonstrated that adolescents’ GAD symptoms and perceived father-adolescent attachment relationship quality bidirectionally negatively affected each other over time. For mothers, adolescents’ GAD symptoms negatively predicted perceived mother-adolescent attachment relationship quality over time. The within-wave correlated residuals between perceived attachment relationship quality with fathers and GAD symptoms were stronger for boys than for girls and stronger for the cohort of middle adolescents than for the cohort of early adolescents. This study demonstrates that both the parents’ and the adolescents’ gender as well as the adolescents’ age affects the relation between adolescents’ GAD symptoms and perceived parent-adolescent attachment relationship quality.

Generalized Anxiety Disorder (GAD) is the most common anxiety disorder in adolescence (Clark et al. 1994; McGee et al. 1990; Whitaker et al. 1990) and is characterized by the experience of excessive, uncontrollable worry over multiple life circumstances (American Psychiatric Association 1994). GAD has an early onset and tends to be chronic (Dugas 2000; Hunt and Singh 1991; Keller et al. 1992). Furthermore, GAD is associated with high rates of comorbidity with other anxiety disorders (Brawman-Mintzer et al. 1993; Brown et al. 2001; Kashani and Orvaschel 1990; Massion et al. 1993; McGee et al. 1990). It is suggested that other anxiety disorders emerge from GAD (Borkovec et al. 2002; Brown et al. 1994). In recent years, research and the treatment related to GAD symptoms has increasingly focused on the individual’s interpersonal functioning (Borkovec et al. 2002; Crits-Christoph 2002). Problems in the parent-adolescent relationship are thought to be a risk factor for the presence and development of GAD symptoms. For instance, the parent-adolescent attachment relationship quality might contribute to the development of GAD symptoms (Behar et al. 2009; Eng and Heimberg 2006; Hale et al. 2006; Viana and Rabian 2008). However, it is also possible that adolescents’ GAD symptoms lead to a lower quality of the parent-adolescent attachment relationship. Moreover, when studying parent-adolescent relationships, the focus predominantly is on mothers and information on the role of the quality of the relationship with fathers is sparse (Bögels and Phares 2008). The current study will therefore examine the bidirectional relation between GAD symptoms and perceived attachment relationship quality with both mothers and fathers.

## Perceived Attachment Relationship Quality and GAD Symptoms

Research on parent-adolescent attachment relationships and adolescents’ anxiety symptoms is critical, since adolescence is a vulnerable period for developing psychosocial problems (Jackson and Goossens 2006), and the parent–child relationship remains important for adolescents’ psychosocial functioning (Laursen and Collins 2009). A main symptom of GAD is excessive, persistent and uncontrollable worry (Comer et al. 2004) and an important aspect of this worry is social-evaluative concerns in interpersonal interactions (Hudson and Rapee 2004). Therefore, adolescent’s perception of interpersonal interaction behaviors in close relationships such as the relationship with parents may be related to adolescents’ symptoms of GAD (Hale et al. 2006).

A few studies examined the specific association between perceived parent-adolescent attachment relationship quality and GAD symptoms in adolescence. Findings revealed that perceived insecure attachment relationships, marked by lower levels of trust, poorer communication, and greater feelings of alienation, were more common in individuals with the diagnosis of GAD than individuals free of this diagnosis (Eng and Heimberg 2006). In addition, GAD symptoms have been associated with perceptions of an insecure attachment in adolescents (Hale et al. 2006) as well as in undergraduate students (Viana and Rabian 2008). More specifically, greater perceptions of alienation have been associated with more severe GAD symptoms (Hale et al. 2006; Viana and Rabian 2008). Thus, perceived quality of the attachment relationship with parents seems to be negatively associated with GAD symptoms.

Generally, these correlational studies tend to interpret the association as evidence for parent effects, demonstrating that parental behavior is influential on the development of adolescent anxiety symptoms (Lollis and Kuczynski 1997). However, the cross-sectional design of these studies does not rule out alternative explanations, such as that GAD symptoms predict a lower quality of the parent-adolescent relationship. To acquire information about the direction of effects between GAD symptoms and perceived relationship quality, full recursive longitudinal models are needed that investigate the bidirectional over-time relation between GAD symptoms and perceived parent-adolescent attachment relationship quality while controlling for concurrent relations at Wave 1, stability, and correlated change of GAD symptoms and attachment relationship. As there are, to the best of our knowledge, no studies yet using these full recursive models on GAD symptoms and perceived attachment relationship quality, the direction of this relation remains unclear. There are a few longitudinal studies that have suggested parent effects, but these studies only investigated the relation between early attachment relationships in infancy and later anxiety symptoms. Findings revealed that an insecure attachment, assessed during infancy, was related to anxiety symptoms in preschool-age children (Shaw et al. 1997), children (Bohlin et al. 2000), and adolescents (Bosquet and Egeland 2006; Warren et al. 1997). Thus, parent-adolescent attachment relationship quality could possibly elicit GAD symptoms. It is possible that a low-quality attachment relationship causes the feeling of not being loved, which in turn can lead to frequently worrying (Buist et al. 2004). Parent effects are also consistent with the suggestion of attachment theory that nonoptimal attachment relationships have implications for the functioning and mental health of adolescents (Bowlby 1977). In sum, although theories support parent effects, the evidence for effects of perceived attachment quality on GAD symptoms in adolescence is at best weak.

In addition to parent effects, it is also possible that characteristics of children have influence on parental behavior (Bell 1968; Lollis and Kuczynski 1997), and that adolescents’ GAD symptoms affect relationship quality with parents. It is conceivable that adolescents with GAD symptoms tend to describe their relationship with parents more negatively over time, because their worrying results in unrealistic perceptions of the relationship and an interpretation of their environment as more dangerous (Buist et al. 2004; Riskind and Williams 2005). It is also possible that it is not just the interpretation of the adolescent that is changing, but that parents’ actual interaction behavior worsens in response to the GAD symptoms of adolescents (Bögels and Brechman-Toussaint 2006), resulting in a more negative perception of the attachment relationship with parents by the adolescent. Thus, instead of or in addition to parent effects, the association between adolescent GAD symptoms and perceived quality of the attachment relationship to parents may be explained by child effects. Perceived attachment relationship quality might affect GAD symptoms and, in turn, adolescents’ GAD symptoms might affect perceived attachment relationship quality. The current study will use a longitudinal, full recursive design to investigate the direction of effects, that is, whether the quality of the attachment relationship with fathers and with mothers predicts GAD symptoms, whether GAD symptoms predict the perceived attachment relationship quality with fathers and mothers, or whether there is a bidirectional effect.

## The Role of Parental and Adolescent Gender and Adolescent Age

Most previous studies have examined associations of GAD with quality of the attachment relationship to both parents as a unit (Eng and Heimberg 2006; Hale et al. 2006; Muris et al. 2001), and fail to make a distinction between attachment relationship quality with fathers and mothers. Nevertheless, the association between perceived attachment relationship quality and adolescents’ GAD symptoms may be different for the relationship with fathers and mothers. It is important to distinguish between fathers and mothers, because research has shown that attachment relationship quality with fathers may have a different impact on various aspects of development and functioning of adolescents than attachment relationship quality with mothers (Liu 2008). Also, a review of the literature on the role of fathers and mothers in child anxiety concluded that father affection and involvement is uniquely and negatively related to anxiety problems in children and adolescents (Bögels and Phares 2008). The only study that distinguished between fathers and mothers in the relation between perceived parent-adolescent attachment relationship quality and GAD symptoms found a significant association between GAD symptoms and attachment relationship quality with mothers, but not with fathers (Viana and Rabian 2008). This difference may be related to the role of parents. Mothers are more involved in daily caretaking than fathers and may therefore be more significant for adolescent psychosocial functioning (Richards et al. 1991). However, an alternative hypothesis may be that as mothers are usually more involved in daily caretaking than fathers, their strong involvement may stand in the way of adolescents’ developmental task of separating from home and developing their autonomy. The involvement of fathers may therefore be more important for adolescents’ separation from the family than the involvement of mothers (Bögels and Phares 2008). We will test these hypotheses by examining whether concurrent and over-time associations of GAD with relationship quality differ for the quality of the attachment relationship with mothers and fathers.

Gender of adolescent may affect associations between perceived attachment relationship quality and GAD symptoms. Girls have a tendency to be more sensitive to interpersonal interactions than boys (Hankin and Abramson 2001; Keijsers et al. 2010). Girls exhibit an increasingly stronger relational orientation and greater emotional needs in adolescence than boys (Rudolph 2002). Therefore, it is assumed that the psychosocial adjustment of girls is more strongly influenced by interpersonal relationships than the psychosocial adjustment of boys (Block 1983). In line with this, Hale et al. (2006) found that the association between adolescent-perceived alienation in the relationship with parents and adolescent GAD symptoms was significantly stronger for mid-adolescent girls (but not early adolescent girls) compared to both early and mid-adolescent boys.

Adolescent age may also play a role in the association between adolescent GAD symptoms and perceived parent-adolescent attachment relationship quality. GAD symptoms have been found to increase from early to middle adolescence (Hale et al. 2005). Parent-adolescent relationships become more equal and horizontal over time and adolescents become increasingly autonomous and independent (De Goede et al. 2009; Russell et al. 1998). The associations between GAD symptoms and perceived attachment relationship quality may become weaker over time, because the influence of parents may lessen as adolescents grow older (Meeus et al. 2005). To the best of our knowledge, age differences in the association between GAD symptoms and perceived parent-adolescent attachment relationship quality have not been investigated yet.

## The Present Study

The purpose of the present study was to investigate the longitudinal, bidirectional relation between perceived attachment relationship quality with fathers and mothers and GAD symptoms in adolescents from age 12 to 20. Additionally, we examined whether gender and age affect this relation. First, this study addressed whether there was a relation between GAD symptoms and perceived parent-adolescent attachment relationship quality over time. More specifically, we examined (1) whether lower levels of perceived attachment relationship quality predict higher levels of GAD symptoms, (2) whether higher levels of GAD symptoms predict lower levels of perceived attachment relationship quality, (3) whether there is a bidirectional effect over time. Since this study is the first that examines the direction of effects in the relation between GAD symptoms and perceived attachment relationship quality, no specific hypothesis of the direction of effects was made. Furthermore, we investigated whether the relation between GAD symptoms and perceived attachment relationship quality differs for adolescent-father dyads and adolescent-mother dyads. Next, we examined whether gender moderates the relation between GAD symptoms and parent-adolescent attachment relationship quality. We hypothesized that the relation will be stronger for girls than for boys. Finally, we examined whether age differences exist in the association between GAD symptoms and perceived attachment relationship quality. We hypothesized that during early adolescence GAD symptoms are more strongly related to perceived parent-adolescent attachment relationship quality than during middle adolescence.

## Method

### Participants

The sample of this study consisted of 1,313 adolescents who participated in the longitudinal project on CONflict And Management Of Relationships (CONAMORE; Meeus et al. 2006). This study used four waves of data (i.e., Wave 1, 3, 4, and 5) with a one-year interval between Wave 3, 4, and 5 and a two-year interval between Wave 1 and 3. Wave 2 was left out, as perceived attachment relationship quality was not assessed during Wave 2. There were no exclusionary criteria with respect to participant selection. The participants came from different Dutch junior high and high schools in the Utrecht province of the Netherlands. Of the 1,313 adolescents, 48.5% were boys and 51.5% were girls. The participants were divided into an early to middle adolescence cohort (*n* = 923) and a middle to late adolescence cohort (*n* = 390). The mean age at the first wave was 12.42 years (*SD* = 0.59) of the early adolescence cohort, and was 16.68 years (*SD* = 0.80) of the middle adolescence cohort. Because both age groups were assessed during five measurement waves, a total age range from 12 to 16 and from 16 to 20 years was available. The majority of the participants were Dutch (82.8%). The rest (17.2%) identified themselves as part of a non-Western ethnic group. The large majority of the adolescents (82.9%) lived with both parents. Sample attrition was 1.2% across waves, and 7.14% of the values was missing across waves. To estimate the pattern of missing values, Little’s (1988) Missing Completely at Random (MCAR) test was conducted. Although this very stringent test was significant, χ^2^(*N* = 1313, df = 411) = 649.54, *p* = 0.00, the χ^2^ ⁄ df ratio of 1.58 indicated a good fit between sample scores with and without imputation (Bollen 1989). Participants with partially missing data could thus be included in the analyses using Full Information Maximum Likelihood in Mplus (Satorra and Bentler 1994).

### Procedure

Adolescents and their parents received written information of the research prior to the study and were given the opportunity to decline from participation. If the adolescent agreed to participate, both adolescent and his or her parents were required to provide written informed consent. Less than 1% chose not to participate. Written informed consent was also obtained for all the participating schools. During annual assessments adolescents completed a series of questionnaires at school after school hours. Confidentiality was guaranteed explicitly. Research assistants gave verbal instructions to the adolescents just before the testing to complement the written instructions printed above each questionnaire. Completing the questionnaires lasted for an hour. Adolescents received €10 as a reward for every wave they participated in.

### Measures

#### GAD Symptoms

GAD symptoms were measured with the GAD subscale of the Screen for Child Anxiety Related Emotional Disorders (SCARED). The SCARED is a self-report questionnaire designed for children and adolescents that measures the occurrence of anxiety disorder symptoms. The GAD subscale consists of 9 items. Example items are: “I worry if others will like me”, and “I worry about the future”. Adolescents rated each statement on a 3-point scale: 0 (almost never), 1 (sometimes), and 2 (often). Items were averaged to compute a mean score, with higher mean scores indicating greater reports of GAD symptoms. The scale has demonstrated good reliability and construct validity (Hale et al. 2005). Reliability (Cronbach’s alpha) in this study ranged from 0.86 to 0.88 over the waves.

#### Perceived Attachment Relationship Quality

The Inventory of Parent and Peer Attachment (IPPA; Armsden and Greenberg 1987) was used to measure the perceived attachment relationship quality. The IPPA is a self-report questionnaire that measures adolescents’ perceptions of their attachment to their parents on three subscales. In this study the questionnaire is completed separately for fathers and mothers. The communication subscale consists of three items. This subscale measures to what extent an adolescent experiences having high quality of communication with parents. An example item is: “If my father/mother knows something is bothering me, he/she asks me.” The trust subscale also contains three items and measures the extent to which an adolescent trusts parents to respect and accept his of her feelings and wishes. An example: “My father/mother respects my feelings.” The alienation subscale contains six items and measures the degree to which an adolescent experiences negative feeling toward parents. For example, the adolescent had to answer the following statement: “My parents have their own problems”. Six-point Likert scales (responses ranging from 1 = ‘never’ to 6 = ‘always’) were used. The overall score for perceived attachment relationship quality is computed by the mean of the three subscales, after recoding the alienation scale. The IPPA proved to be reliable and valid in previous studies (Armsden and Greenberg 1987; Deković and Meeus 1997; Raja et al. 1992; Vivona 2000). Cronbach’s alphas of the subscales in this study ranged from 0.74 to 0.91. Reliability across waves for the overall perceived attachment relationship quality score ranged from 0.83 to 0.87 for fathers and from 0.84 to 0.86 for mothers.

### Data Analysis

The research questions are examined with cross-lagged path analyses within Mplus Version 5 (Muthén and Muthén 2007). Data were analyzed for fathers and mothers separately.

The first step in the analyses was testing a baseline model for the total sample, in which we estimated stability paths (autocorrelations) of GAD symptoms, stability paths of perceived attachment relationship quality, as well as correlations between within-wave residuals of GAD symptoms and perceived attachment relationship quality. In subsequent models cross-lagged paths were added to the baseline model to investigate whether this improved the overall fit of the total model. The second model was a unidirectional model from adolescents’ GAD symptoms to perceived attachment relationship quality, implying that GAD symptoms in adolescents affect perceived parent-adolescent attachment relationship quality and not vice versa. The third model was also a unidirectional model, but in this model paths were drawn from perceived attachment relationship quality to GAD symptoms, implying that adolescents’ perception of attachment relationship quality with parents affect adolescents’ GAD symptoms and not vice versa. The last model was a bidirectional model with mutual paths between GAD symptoms and perceived attachment relationship quality, based on the assumption that there are bidirectional effects between these constructs. For the sake of parsimony we tested whether it was possible to fix over the waves the within-wave correlated residuals and the cross-lagged paths between adolescents’ GAD symptoms and perceived parent-adolescent attachment relationship quality.

After determining which model best represented the data of the total sample, we used two series of multigroup analyses to test the moderation effects of gender and age on the associations between GAD symptoms and perceived attachment relationship quality. We constrained within-wave correlated residuals and cross-lagged paths between adolescents’ GAD symptoms and perceived attachment relationship quality to be equal across groups (i.e., boys vs. girls, early cohort vs. middle cohort). When fixation of different paths to be equal significantly impaired the model fit, there were significant differences between the two groups, and we released the constraints again.

For evaluating the fit of the models, several goodness-of-fit indices were used: the Comparative Fit Index (CFI), with values above 0.90 representing a satisfactory fit and values above 0.95 demonstrating a good fit; the Root Mean Square Error of Approximation (RMSEA), with values up to 0.10 indicating an acceptable fit and values up to 0.05 being indicative of a good fit (Hu and Bentler 1999). Chi-square difference tests were used to compare the fit of different models. Relatively lower RMSEA’s and higher CFI’s also indicate better fit (Kline 2010).

## Results

### Descriptive Statistics

Table 1 provides the means and standard deviations of perceived attachment relationship quality and GAD symptoms for each gender and age cohort at Wave 1, 3, 4, and 5. Repeated measures ANOVAs demonstrated a significant main effect of gender for perceived attachment quality of relationship with mothers and GAD symptoms. Girls reported more GAD symptoms and higher quality of attachment relationship with mothers than boys. A significant main effect of age cohort was found for GAD symptoms. Early adolescents reported less GAD symptoms in comparison with middle adolescents. For perceived attachment relationship quality with both fathers and mothers, a significant interaction effect between age cohort and waves was found, indicating that perceived attachment relationship quality declined from early to middle adolescence and increased from middle to late adolescence. Correlation coefficients between perceived attachment relationship quality with fathers and mothers and GAD symptoms are reported in Table 2.

**Table 1:** Mean age and gender differences in perceived attachment relationship quality with fathers and mothers and GAD symptoms

Variable source	Attachment quality fathers		Attachment quality mothers		GAD symptoms	
	*M*	*SD*	*M*	*SD*	*M*	*SD*
Wave 1						
Boys (*n* = 637)	4.07	0.82	4.26	0.91	1.31	0.39
Girls (*n* = 676)	4.15	0.81	4.58	0.85	1.43	0.41
Early cohort (*n* = 923)	4.20	0.83	4.46	0.91	1.34	0.39
Middle cohort (*n* = 390)	3.95	0.77	4.35	0.85	1.43	0.41
Wave 3						
Boys (*n* = 637)	4.07	0.76	4.25	0.73	1.29	0.35
Girls (*n* = 676)	4.13	0.84	4.52	0.79	1.46	0.43
Early cohort (*n* = 923)	4.08	0.81	4.36	0.78	1.35	0.39
Middle cohort (*n* = 390)	4.14	0.79	4.47	0.75	1.44	0.42
Wave 4						
Boys (*n* = 637)	4.13	0.76	4.33	0.75	1.27	0.33
Girls (*n* = 676)	4.16	0.86	4.49	0.81	1.49	0.44
Early cohort (*n* = 923)	4.13	0.82	4.38	0.79	1.35	0.40
Middle cohort (*n* = 390)	4.18	0.80	4.50	0.76	1.44	0.43
Wave 5						
Boys (*n* = 637)	4.18	0.76	4.38	0.71	1.25	0.32
Girls (*n* = 676)	4.20	0.85	4.54	0.78	1.49	0.45
Early cohort (*n* = 923)	4.18	0.82	4.44	0.76	1.35	0.39
Middle cohort (*n* = 390)	4.21	0.76	4.53	0.74	1.44	0.45
*F*gender(1,979)	1.23		28.14**		82.23**	
*F*agecohort(1,979)	1.62		0.12		8.31**	
*F*time(1,979)	4.79**		2.83*		0.37	
*F*time*agecohort(1,979)	13.99**		9.53**		0.21	

*p < 0.05. **p < 0.01

**Table 2:** Correlation coefficients between perceived attachment relationship quality with fathers and mothers and GAD symptoms

	Attachment quality fathers				Attachment quality mothers				GAD symptoms			
	Wave 1	Wave 3	Wave 4	Wave 5	Wave 1	Wave 3	Wave 4	Wave 5	Wave 1	Wave 3	Wave 4	Wave 5
Attachment quality fathers												
Wave 1												
Wave 3	0.47**											
Wave 4	0.41**	0.65**										
Wave 5	0.34**	0.58**	0.68**									
Attachment quality mothers												
Wave 1	0.64**	0.25**	0.20**	0.16**								
Wave 3	0.32**	0.55**	0.41**	0.36**	0.46**							
Wave 4	0.24**	0.40**	0.59**	0.42**	0.34**	0.61**						
Wave 5	0.21**	0.35**	0.45**	0.58**	0.29**	0.54**	0.68**					
GAD symptoms												
Wave 1	−0.18**	−0.15**	−0.20**	−0.19**	−0.13**	−0.14**	−0.20**	−0.16**				
Wave 3	−0.15**	−0.23**	−0.23**	−0.22**	−0.12**	−0.19**	−0.19**	−0.20**	0.49**			
Wave 4	−0.11**	−0.17**	−0.22**	−0.24**	−0.08*	−0.13**	−0.18**	−0.18**	0.42**	0.67**		
Wave 5	−0.10**	−0.18**	−0.19**	−0.24**	−0.04	−0.10**	−0.15**	−0.19**	0.45**	0.61**	0.72**	

*p < 0.05. **p < 0.01.

### Perceived Attachment Relationship Quality and GAD Symptoms

For all adolescents, we tested whether adding the cross-lagged paths between GAD symptoms and perceived attachment relationship quality (Model 2, 3, and 4) improved model fit compared to a baseline model with only stability paths and within-wave correlated residuals (Model 1). For fathers, Models 2, 3, and 4 significantly improved model fit compared to the baseline model (Model 1). So we continued with Model 4 that has stability paths, within-wave correlated residuals and cross-lagged paths from GAD symptoms to perceived attachment relationship quality, as well as cross-lagged paths from perceived attachment relationship quality to GAD symptoms (Table 3). We tested whether it was possible to fix over the waves the within-wave correlated residuals and the cross-lagged paths. Paths could be constrained to be equal across waves from GAD symptoms to perceived attachment relationship quality, Δ*χ*SB*²* (2, *N* = 1313) = 0.54, *p* = 0.76, and from perceived attachment relationship quality to GAD symptoms, Δ*χ*SB*²* (2, *N* = 1313) = −3.64, *p* = 1.00. Note that although unstandardized coefficients were constrained to be equal, the standardized coefficients can still slightly differ from each other. It was not possible to constrain the paths from GAD symptoms to perceived attachment relationship quality to be equal to the paths from perceived attachment relationship quality to GAD symptoms, Δ*χ*SB*²* (1, *N* = 1313) = 34.84, *p* < 0.01. It was also not possible to fix the within-wave correlated residuals across waves, Δ*χ*SB*²* (2, *N* = 1313) = 8.19, *p* < 0.05. Model fit for the final model for fathers was: *χ*SB*²* (16) = 182.17, *p* = 0.80; CFI = 0.94; RMSEA = 0.09.

**Table 3:** Model comparisons of cross-lagged path analysis of GAD symptoms and perceived attachment relationship quality with fathers and mothers

	Model fit indices				Model comparison test		
	χSB²	*df*	CFI	RMSEA		Δ*χ*SB²	Δ*df*
Fathers							
Model 1. Baseline model	228.17	18	0.92	0.09			
Model 2. Baseline+paths GAD symptoms → attachment quality	186.70	15	0.94	0.09	2 vs. 1	40.93**	3
Model 3. Baseline+paths attachment quality → GAD symptoms	214.35	15	0.93	0.10	3 vs. 1	18.07**	3
Model 4. Baseline+bidirectional paths	173.28	12	0.94	0.10	4 vs. 1	54.89**	6
					4 vs. 2	9.13*	3
					4 vs. 3	40.69**	3
Mothers							
Model 1. Baseline model	189.33	18	0.94	0.09			
Model 2. Baseline+paths GAD symptoms → attachment quality	168.04	15	0.94	0.09	2 vs. 1	20.32**	3
Model 3. Baseline+paths attachment quality → GAD symptoms	181.97	15	0.94	0.09	3 vs. 1	4.39	3
Model 4. Baseline+bidirectional paths	160.45	12	0.94	0.10	4 vs. 1	24.29**	6
					4 vs. 2	3.79	3
					4 vs. 3	19.72**	3

*p < 0.05. **p < 0.01

For mothers, both Models 2 (baseline model with paths from GAD symptoms to attachment quality) and 4 (baseline model with bidirectional paths) improved fit compared to the baseline model (Model 1). As Model 3 (baseline model with paths from attachment quality to GAD symptoms) did not improve fit compared to the baseline model, and Model 4 did not improve fit compared to Model 2, we chose Model 2 as the best fitting model (Table 3). Paths could be constrained to be equal across waves from GAD symptoms to perceived attachment relationship quality, Δ*χ*SB*²* (2, *N* = 1313) = 1.63, *p* = 0.44. It was also possible to fix the within-wave correlated residuals, Δ*χ*SB*²* (2, *N* = 1313) = 4.02, *p* = 0.13. Model fit for the final model for mothers was: *χ*SB*²* (19) = 177.67, *p* = 0.22; CFI = 0.94; RMSEA = 0.08.

Results show that initial correlations between relationship quality and GAD symptoms were small for the attachment relationship quality with mothers and small to moderate for the attachment relationship quality with fathers (see Table 4). The within-wave correlated residuals at Wave 3 to 5 were small to moderate for both parents, showing that relative change in attachment relationship quality is negatively associated with relative change in GAD symptoms. Perceived attachment relationship quality with fathers significantly predicted later GAD symptoms (ß = −0.04). Lower level of perceived father-adolescent attachment relationship quality was related to more GAD symptoms later in life. In addition, GAD symptoms significantly predicted later perceived attachment relationship quality with both parents (ß = −0.06 to -0.09). More GAD symptoms were related to lower levels of perceived attachment relationship quality with parents later in life.

**Table 4:** Cross-lagged path analyses of perceived attachment relationship quality with fathers and mothers and GAD symptoms

Parent	Fathers		Mothers	
	B¹	ß	B	ß
Stability paths				
GAD symptoms W1→W3	0.49 (0.42, 0.56)	0.49**	0.49 (0.42, 0.56)	0.50**
GAD symptoms W3→W4	0.68 (0.62, 0.73)	0.66**	0.68 (0.63, 0.73)	0.67**
GAD symptoms W4→W5	0.72 (0.66, 0.77)	0.71**	0.73 (0.68, 0.78)	0.72**
Attachment quality W1→W3	0.44 (0.38, 0.51)	0.45**	0.39 (0.34, 0.44)	0.45**
Attachment quality W3→W4	0.64 (0.59, 0.70)	0.63**	0.61 (0.56, 0.67)	0.60**
Attachment quality W4→W5	0.67 (0.62, 0.72)	0.67**	0.65 (0.61, 0.70)	0.68**
Initial correlation				
Attachment quality W1 – GAD symptoms W1	−0.06 (−0.08, −0.04)	−0.18**	−0.05 (−0.07, -0.02)	−0.13**
Within-wave correlated residuals				
Attachment quality W3 – GAD symptoms W3	−0.04 (−0.06,−0.02)	−0.17**	−0.02 (−0.03, -0.01)	−0.09**
Attachment quality W4 – GAD symptoms W4	−0.02 (−0.03, -0.00)	−0.09**	−0.02 (−0.03, -0.01)	−0.11**
Attachment quality W5 – GAD symptoms W5	−0.01 (−0.03, -0.00)	−0.08*	−0.02 (−0.03, -0.01)	−0.14**
Cross-lagged effects				
GAD symptoms W1 → Attachment quality W3	−0.18 (−0.24, -0.12)	−0.09**	−0.12 (−0.18, -0.07)	−0.06**
GAD symptoms W3 → Attachment quality W4	−0.18 (−0.24, -0.12)	−0.09**	−0.12 (−0.18, -0.07)	−0.06**
GAD symptoms W4 → Attachment quality W5	−0.18 (−0.24, -0.12)	−0.09**	−0.12 (−0.18, -0.07)	−0.07**
Attachment quality W1 → GAD symptoms W3	−0.02 (−0.03, -0.01)	−0.04**	–	–
Attachment quality W3 → GAD symptoms W4	−0.02 (−0.03, -0.01)	−0.04**	–	–
Attachment quality W4 → GAD symptoms W5	−0.02 (−0.03, -0.01)	−0.04**	–	–

*p < 0.05. **p < 0.01

¹Confidence intervals for B’s are displayed between brackets

### Gender Differences

Using multigroup analyses with boys and girls as the two groups, we tested whether the within-wave correlated residuals and cross-lagged paths between GAD symptoms and perceived attachment relationship quality could be constrained to be equal across groups.

For perceived attachment relationship quality with fathers, the chi-square difference tests showed that the model with different paths for the groups provided a better fit to the data than the model with equality constraints across groups for within-wave correlated residuals, Δ*χ*SB*²* (1, *N* = 1313) = 21.86, *p* < 0.05. The models with different paths from GAD symptoms to perceived attachment relationship quality, Δ*χ*SB*²* (1, *N* = 1313) = 0.63, *p* = 0.43, and with different paths from perceived attachment relationship quality to GAD symptoms, Δ*χ*SB*²* (1, *N* = 1313) = 0.36, *p* = 0.55, did not provide a better fit. Model fit for the final model for fathers was: *χ*SB*²* (34) = 201.98, *p* = 0.63; CFI = 0.94; RMSEA = 0.09.

For adolescents’ perceived attachment relationship quality with mothers, the model with different paths for the groups did not provide a better fit for the data than the model with equality constraints across groups for within-wave correlated residuals, Δ*χ*SB*²* (1, *N* = 1313) = 0.85, *p* = 0.35, and for paths from GAD symptoms to perceived attachment relationship quality, Δ*χ*SB*²* (1, *N* = 1313) = 0.03, *p* = 0.98. Model fit for the final model for mothers was: *χ*SB*²* (41) = 198.97, *p* = 0.84; CFI = 0.94; RMSEA = 0.08.

Thus, there were only gender differences in the within-wave correlated residuals between GAD symptoms and perceived attachment relationship quality with fathers. These correlations were stronger for boys (ß’s ranged from −0.12 to −0.20) than for girls (ß’s ranged from −0.05 to −0.17).

### Age Cohort Differences

Using multigroup analyses with the two age cohorts as the two groups, we tested whether the within-wave correlated residuals and cross-lagged paths between GAD symptoms and perceived attachment relationship quality could be fixed across groups. For perceived attachment relationship quality with fathers, the chi-square difference tests showed that the model with different paths for the two age cohorts provided a better fit to the data than the model with equality constraints across groups for within-wave correlated residuals, Δ*χ*SB*²* (1, *N* = 1313) = 21.64, *p* < 0.05. The models with different paths from GAD symptoms to perceived attachment relationship quality, Δ*χ*SB*²* (1, *N* = 1313) = 0.74, *p* = 0.39, and with different paths from perceived attachment relationship quality to GAD symptoms, Δ*χ*SB*²* (1, *N* = 1313) = −0.11, *p* = 1.00, did not provide a better fit. Model fit for the final model for fathers was: (34) = 212.96, *p* = 0.66; CFI = 0.94; RMSEA = 0.09.

For adolescents’ perceived attachment relationship quality with mothers, the model with different paths for the groups did not provide a better fit for the data than the model with equality constraints across groups for within-wave correlated residuals, Δ*χ*SB*²* (1, *N* = 1313) = 0.45, *p* = 0.80, and for paths from GAD symptoms to perceived attachment relationship quality, Δ*χ*SB*²* (1, *N* = 1313) = 2.80, *p* = 0.09. Model fit for the final model for mothers was: *χ*SB*²* (41) = 217.02, *p* = 0.35; CFI = 0.94; RMSEA = 0.08.

**Fig. 1:**
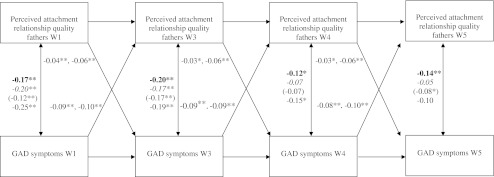
Relation between Perceived Atachment Relationship Quality with Fathers and GAD Symptoms. *Note.* For the cross-lagged paths, the range of ß’s is shown for gender and age cohort (these paths could be constrained to be equal). For the within-wave correlated residuals, the ß’s are shown separately for each group. Bold = boys, italic = girls, between brackets = early adolescent cohort, normal = middle adolescent cohort. *p < 0.05. **p < 0.01

**Fig. 2:**
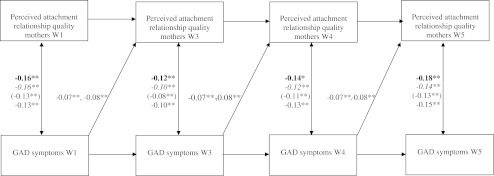
Relation between Perceived Attachment Relationship Quality with Mothers and GAD Symptoms. *Note.* For the cross-lagged paths, the range of ß’s is shown for gender and age cohort (these paths could be constrained to be equal). For the within-wave correlated residuals, the ß’s are shown separately for each group. Bold = boys, italic = girls, between brackets = early adolescent cohort, normal = middle adolescent cohort. *p < 0.05. **p < 0.01

Thus, the only difference between the early and middle adolescents cohort was in the within-wave correlated residuals between GAD symptoms and perceived attachment relationship quality with fathers. These correlations were stronger for the cohort of middle adolescents (ß’s ranged from −0.10 to −0.19) than for the cohort of early adolescents (ß’s ranged from −0.07 to −0.17). Figures 1 and 2 present the graphical models of the relations between GAD symptoms and perceived attachment relationship quality with fathers and mothers.

## Discussion

The main aims of this study were to investigate the bidirectional relation between perceived attachment relationship quality with both fathers and mothers and GAD symptoms over time, and to examine whether these associations differed for boys and girls, and early and middle adolescents. The results showed a bidirectional relation between GAD symptoms and perceived quality of attachment relationship with fathers, whereas a unidirectional model was found for mothers. Only adolescents’ GAD symptoms longitudinally predicted perceived mother-adolescent attachment relationship quality. Gender and age moderated the within-wave correlated residuals between GAD symptoms and attachment relationship quality with fathers, but not with mothers.

### Perceived Attachment Relationship Quality and GAD Symptoms

The most consistent evidence was found for child effects: Adolescents who reported higher levels of GAD symptoms perceived lower quality of attachment relationship with both fathers and mothers one or two years later. This finding can be interpreted in two ways. A possibility is that parents of adolescents with self-reported GAD symptoms change their behaviors toward their adolescent children. It is assumed that emotional problems of adolescents could lead to negative responses of their parents (Bögels and Brechman-Toussaint 2006). A second possibility is that adolescents no longer can see their relationship with their parents as positive due to their anxiety symptoms (Buist et al. 2004). Individuals with GAD symptoms have a dysfunctional cognitive style. They have the tendency to generate fearful mental representations and predictions of escalating risk and danger, and may over time interpret their environment as more threatening (Riskind and Williams 2005). Thus, the effects of GAD on perceived attachment relationship quality with both parents may be a sign of real parenting interactions or may reflect the adolescents’ interpretation of the relationship.

In addition to the child effects, parent effects were found for the attachment relationship with fathers, but not for the attachment relationship with mothers. Adolescents who perceived lower quality of attachment relationship with fathers reported higher levels of GAD symptoms one or two years later. This finding supports attachment theory that proposes that parents foster healthy development among adolescents (Bowlby 1977). In addition, based on the assumption that a lower attachment relationship quality is associated with the idea of adolescents of not being loved, adolescents develop internalizing problems (Buist et al. 2004). However, paths from GAD symptoms to perceived father-adolescent attachment relationship quality were stronger than paths from perceived father-adolescent attachment relationship to GAD symptoms. So, although the evidence for parent effects is consistent with theory, this evidence was less consistent than that for child effects.

Although we found a difference between GAD and attachment relationship quality with fathers and mothers, it was not the difference that was expected. We hypothesized that the relation between GAD symptoms and perceived attachment relationship quality would be found for mothers but not for fathers (Liu 2008; Richards et al. 1991; Viana and Rabian 2008), yet findings revealed that only the attachment relationship quality with fathers predicted GAD symptoms, suggesting that fathers play an important role in the development of adolescents. This is consistent with findings that fathers have a unique, substantial impact on the psychological well-being of adolescents, in addition to the influence that mothers have (Videon 2005). The finding is also in line with the suggestion that adolescents have the developmental task to separate from their families, and as mothers are usually more involved in daily caretaking, the involvement of a father might be crucial for adolescents to separate from home and develop further autonomy (Bögels and Phares 2008). Another possibility is that fathers are generally less involved in the adolescents’ life and may have a less unconditional positive attitude toward their children compared to mothers, and therefore inter-individual differences among fathers are more important. Replication of these findings is warranted, but it is obvious that fathers should be involved in future research and treatment of GAD symptoms.

Besides the longitudinal relation between GAD symptoms and perceived parent-adolescent attachment relationship quality, there were also within-wave correlated residuals between those constructs. In accordance with the findings of Hale et al. (2006) and Viana and Rabian (2008), higher levels of GAD symptoms were associated with lower perceived quality of attachment relationship with parents. The initial correlation between relationship quality and GAD symptoms was small to moderate for the attachment relationship quality with fathers and small for mothers. The within-wave correlated residuals were small to moderate across waves for both parents. These correlations suggest that an increase in attachment relationship quality is related to a decrease in GAD symptoms.

### Differences Between Boys and Girls

We investigated whether the relation between perceived attachment relationship quality with fathers and mothers and GAD symptoms differed for boys and girls. We expected that this relation would be stronger for girls than boys, because girls have a stronger relational orientation (Rudolph 2002) and a genetic vulnerability to anxiety (Silberg et al. 2001). However, no differences were found in the cross-lagged paths between perceived attachment relationship quality with both parents and GAD symptoms. Gender only affected the within-wave correlated residuals between adolescents’ GAD symptoms and perceived father-adolescent attachment relationship quality. These correlations were somewhat stronger for boys than for girls. This small difference can be explained by the fact that sons are more emotionally involved with their fathers than daughters are (Harris et al. 1998). Moreover, same-gender attachment relationships are generally of higher quality than different-gender attachment relationships during adolescence (Buist et al. 2002). However, future research on the interplay between parent and adolescent gender regarding the relation between GAD symptoms and attachment relationship quality is necessary.

### Age Cohort Differences

Besides adolescent gender differences, we also examined the moderating role of age. No differences were found between the cohort of early adolescents and the cohort of middle adolescents for the cross-lagged paths. Age only moderated the within-wave correlated residuals between adolescents’ GAD symptoms and perceived father-adolescent attachment relationship quality. Contrary to what was expected, we found that the correlations for middle to late adolescents were slightly stronger than for early to middle adolescents. This finding can possibly be explained by the fact that GAD symptoms in adolescence increase (Hale et al. 2005). In addition, it may be that fathers are more important for psychosocial functioning of older adolescents, for whom the separation from the family may be more salient (Bögels and Phares 2008) and for whom the instrumental aspects of caregiving characteristic for fathers become more relevant (Richards et al. 1991). Further research is needed to replicate these findings.

### Strengths and Limitations

The current study has several important strengths. Firstly, we extended current knowledge of the relationship between adolescents’ GAD symptoms and perceived attachment relationship quality by using a longitudinal design. This allowed us to disentangle the direction of paths between GAD symptoms and perceived parent-adolescent attachment relationship quality. Previous studies investigating this association were cross-sectional. Many studies still rely on a parent effect model, but our study confirms that it is important to take account of child effects. Furthermore, the relation between GAD symptoms and perceived attachment relationship quality was examined separately for fathers and mothers. This is an important strength as fathers are generally neglected in research on adolescent anxiety (Bögels and Phares 2008). Our findings suggest that the influence of fathers and mothers on the adjustment of adolescents is different. However, more research is needed to replicate these findings.

Several limitations should also be mentioned. First, the present study only included adolescents from the general population. Because of this, our study is not comparable with studies that gathered data from adolescents diagnosed with GAD. However, research on this population can give insight into issues relevant to the clinical setting. Moreover, since the sample consisted predominantly of Dutch adolescents, the results of the present study cannot be generalized to other samples of adolescents. Furthermore, although the longitudinal design of our study gives insight in the direction of effects, no causal conclusions can be drawn. Another caveat of the current study is the use of adolescents’ self-reports to assess the quality of the parent-adolescent attachment relationship. It is not certain that parents’ actual behavior exactly corresponds to the adolescent’s perception of parental behaviors. Additionally, parents’ and adolescents’ perceptions of the quality of their attachment relationship often diverge (Renk et al. 2008). The use of adolescents’ self-reports regarding perceived attachment relationship quality is justified, because the subjective experience of being ‘brought up’ has more influence on the development of adolescents than parents’ perception of their behaviors (Steinberg et al. 1992). Nevertheless, using observations of actual parental behaviors can expand knowledge of the relationship between attachment relationship quality and GAD symptoms. With respect to self-reports of GAD symptoms, it is generally accepted that adolescents should be the main informants in the case of anxiety disorders (Stallings and March 1995). However, multi-informant questionnaires could give more information about this relation. This should be addressed in future studies. Finally, we should note that although we found significant results, the absolute value of these betas was small. The large size of our sample might have influenced the significance of correlations and beta values. However, small effects can be important if they are theoretically meaningful (Prentice and Miller 1992). The small cross-lagged effects were found while controlling for the ongoing relation between perceived attachment quality and GAD symptoms, and we think our findings have the potential to make a meaningful contribution to the field.

## Conclusion

In sum, the results of the present study indicate that adolescents’ GAD symptoms and perceived parent-adolescent attachment relationship quality are longitudinally associated. The direction of effects depends on parents’ gender. GAD symptoms consistently affected later perceived attachment relationship quality with both parents. Only for fathers, the perceived quality of attachment relationship predicted later GAD symptoms. However, child effects were stronger. Furthermore, our results revealed that the within-wave correlated residuals between GAD symptoms and perceived father-adolescent attachment relationship were stronger for boys than for girls, and stronger for middle adolescents than for early adolescents. So, there is a longitudinal, negative relation between GAD symptoms and perceived parent-adolescent attachment relationship that depends on the interplay between parent and adolescent gender an adolescent age. Prevention and treatment programs for GAD symptoms should focus on how these symptoms affect adolescents’ perception of the attachment relationship quality with parents. Moreover, our results show the importance of encouraging paternal involvement in adolescents, as well as the importance of involving fathers, in addition to mothers, in research and treatment of GAD symptoms in adolescence.
